# Primary Indolent Acute Promyelocytic Leukemia

**DOI:** 10.3390/hematolrep18010012

**Published:** 2026-01-27

**Authors:** Breanne Wolfenbarger, Daley Morera, Brandol Wolfenbarger, Anand Jillella, Mei Zheng

**Affiliations:** 1 Alabama College of Osteopathic Medicine, Dothan, AL 36303, USA; wolfenbargerb4335@acom.edu; 2 Department of Pathology, Medical College of Georgia, Augusta University, Augusta, GA 30912, USA; dmorera@augusta.edu (D.M.); bwolfenbarger@augusta.edu (B.W.); 3 Department of Medicine, Hematology and Oncology, Medical College of Georgia, Augusta University, Augusta, GA 30912, USA; ajillella@augusta.edu

**Keywords:** acute promyelocytic leukemia, *PML::RARA*, indolent APL

## Abstract

**Background and Clinical Significance:** Acute promyelocytic leukemia (APL) is a rapidly progressive subtype of acute myeloid leukemia defined by *PML::RARA* fusion and characterized by life-threatening coagulopathy. Because the disease typically follows an aggressive course, immediate treatment is essential once APL is suspected. This case report describes an atypical de novo presentation marked by indolent progression rather than the expected aggressive trajectory. **Case Presentation:** A 37-year-old female exhibited gradually declining white blood cell and neutrophil counts over the course of a year, followed by unexplained pancytopenia with severe neutropenia (0.1 × 10^9^/L). Evaluation for nutritional deficiencies and autoimmune disease was unrevealing aside from a positive ANA without clinical features of autoimmunity. Bone-marrow biopsy demonstrated morphologic and flow cytometric findings suggestive of APL, low-level t(15;17), *PML::RARA* fusion, and concomitant *TP53* loss and *ETV6* mutation. Despite the indolent clinical presentation and low disease burden, the molecular and cytogenetic findings confirmed the diagnosis of classical APL with *TP53* loss and *ETV6* mutation. Induction therapy with all-trans-retinoic acid and arsenic trioxide resulted in hematologic remission. **Conclusions:** This case highlights an unusually indolent form of de novo APL not previously documented in the literature, expanding the recognized clinical spectrum of the disease. The findings emphasize the importance of still considering severe diagnoses, such as APL, when presentations deviate from classical patterns. Atypical clinical trajectories should prompt careful assessment of marrow morphology and immunophenotypic features. Continued characterization of such cases may refine diagnostic criteria and direct individualized approaches to therapy.

## 1. Introduction

Acute promyelocytic leukemia (APL) is a rare subtype of acute myeloid leukemia (AML), accounting for 7% to 8% of adult AML cases, and is characterized by a specific chromosomal translocation, t(15;17)(q24.1;q21.2), which generates the *PML::RARA* fusion gene [1]. APL often presents as generalized weakness and fatigue, with bleeding tendencies such as petechiae and gingival bleeding [2].

Historically, it has been considered a hematologic emergency, associated with disseminated intravascular coagulation and hemorrhage, and rapid progression to multi-organ failure if not promptly diagnosed and treated. Recognizing a patient’s symptoms, along with reviewing an emergency CBC and blood smear, is key to identifying abnormal blasts or promyelocytes and initiating the appropriate diagnostic work-up. The first effective treatment for APL was anthracycline, which was introduced in 1973, followed by all-trans-retinoic acid (ATRA) in the 1980s, which improved response rates but not duration of response [3]. In the modern era, APL has become a highly curable subtype of acute myeloid leukemia (AML), with a combination of ATRA plus arsenic trioxide (ATO) achieving complete remission rates approaching 100% and event-free survival exceeding 90% [1,3]. In contrast, AML overall continues to be associated with substantially poorer outcomes based on population statistics, demonstrating a 5-year relative survival of 32.9% [4].

Despite the improvement in survival rate, APL relapses and treatment-resistant cases remain a challenge. An additional challenge is differentiation syndrome, which is characterized by fever, dyspnea, hypotension, weight gain, pleural or pericardial effusions, and acute renal failure, occurs in 20–25% of treated patients, posing a potentially life-threatening complication [2,5]. To our knowledge, indolent de novo APL has not previously been described. Here, we report on the clinical course, diagnosis, and treatment of a patient with indolent de novo APL.

## 2. Case Presentation

A 37-year-old female with no significant medical history was referred in June 2025 for evaluation of intermittent neutropenia and reported gradually worsening pancytopenia. The patient endorsed a longstanding history of easy bruising “for years” but reported no active infections or other systemic symptoms. Historical records revealed that her first documented episode of neutropenia occurred three years earlier, on May 20, 2022, when incidental laboratory testing showed a white blood cell count (WBC) of 0.9 × 10^9^/L, absolute neutrophil count (ANC) of 0.3 × 10^9^/L, hemoglobin 11.1 g/dL, hematocrit 32.9%, and platelets 134 × 10^9^/L. She was discharged from the emergency department with instructions to follow up with hematology, though no subsequent evaluations from that period were available for review. A comprehensive blood count (CBC) from mid-2024 was unremarkable, with a WBC of 7.0 × 10^9^/L, suggesting at least intermittent normalization of counts with a reported gradual decline from there, though interval labs were not available.

Her next documented abnormalities appeared on May 27, 2025, when a routine CBC demonstrated a WBC of 1.1 × 10^9^/L, lymphocytes 63.3%, granulocytes 27.9%, hemoglobin 11.9 g/dL, hematocrit 35.9%, MCV 106.4 fL, MCH 35.3 pg, and platelets 137 × 10^9^/L. At that time, the patient reported night sweats, fatigue, and dry cough at night for one week.

On 10 June 2025, she was evaluated by hematology/oncology for new-onset cytopenias. Her CBC showed a WBC of 1.0 × 10^9^/L, ANC 0.2 × 10^9^/L, hemoglobin 10.5 g/dL, and platelets 271 × 10^9^/L. Coagulation studies at this time showed PT, INR, and PTT within normal limits. Fibrinogen was decreased at 178 mg/dL and D-dimer was elevated at 845 ng/mL, a pattern consistent with the coagulopathy commonly observed in APL. Two weeks later, platelets had declined sharply to 71 × 10^9^/L, while hemoglobin remained stable. Evaluation for nutritional, infectious, and autoimmune etiologies was unrevealing aside from a positive ANA without clinical features of autoimmune disease.

**Figure 1:**
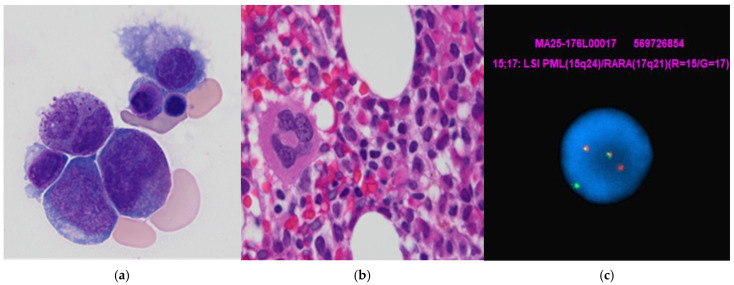
Morphologic and cytogenetic study of APL case with indolent behavior in a treatment naïve individual: (**a**) bone-marrow aspirate showing APL cells with “figure of 8” nuclear morphology, smooth chromatin, and cytoplasmic hot pink granules (×1000); (**b**) bone-marrow core biopsy demonstrates increased promyelocytes/blasts in a background of decreased trilineage maturing hematopoiesis (×1000); (**c**) *PML::RARA* FISH study shows *PML::RARA* fusion signal in yellow (*PML*-red/*RARA*-green).

**Figure 2:**
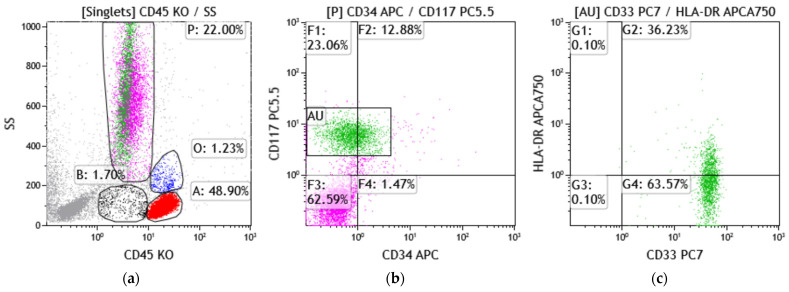
(**a**–**c**) Bone-marrow aspirate flow cytometry study dot plot shows typical APL immunophenotype (CD34−/CD117+/HLA-DR predominantly−; population of interest in green).

Despite a nondiagnostic peripheral smear showing no definitive circulating blasts and a limited aspirate showing an elevated blast count of 5.7% (normal 0–3%), a bone-marrow biopsy, performed on 25 June 2025, revealed a normocellular marrow with increased immature cells exhibiting irregular nuclear contours, relatively smooth chromatin, and cytoplasmic hot-pink granules (Figure 1a,b). Immunostaining demonstrated diffuse CD117 and myeloperoxidase positivity, with focal CD34-positive, increased p53-positive myeloblasts with a background of CD34-negative and p53-negative immature cells. Flow cytometric immunophenotyping (Figure 2a) did not demonstrate a readily identifiable abnormal immunophenotype on screening dot plots; however, detailed analysis identified a small discrete population of CD117-positive immature myeloid cells (Figure 2b) with absent CD34 and negative to rare partial HLA-DR expression (Figure 2b,c), a classical immunophenotype consistent with promyelocytes. Other rare, unusual APL immunophenotypes include (A) CD34+ CD2+ APL, frequently HLA-DR−, and more commonly seen in microgranular variants and variant RARA rearrangements [6]. These cases can mimic non-APL AML and cause diagnostic delay; (B) HLA-DR + APL, often associated with variant RARA fusions and/or therapy-related APL; (C) CD56+ APL, often associated with higher relapse risk and poorer prognosis in some studies [7]; (D) CD11b+ or CD15+ APL, suggested myeloid maturation and seen in APL with differentiation or post-therapy or atypical cases; and (E) basophilic differentiation, manifested by the expression of CD203c and/or CD22, which has been reported in up to one-third of APL cases at presentation and is also observed in patients after ATRA and/or ATO therapy [8].

Conversely, immunophenotypes of some AML cases can also mimic APL, and express strong CD33 with negative CD34 and HLA-DR. These AMLs include *NPM1*-mutated AML; *FLT3*-ITD–positive AML, and AML with monocytic differentiation [8,9].

Cytogenetic analysis identified t(15;17) in approximately 10% of examined cells and 17p loss (*p53*). Next-generation sequencing detected a low-level *ETV6* frameshift mutation, and molecular testing confirmed the *PML::RARA* fusion, establishing the diagnosis of a classical APL with poor prognostic molecular features (Figure 1c). Based on her presented WBC and platelet count, she was classified as low-risk APL.

The patient began ATRA on 27 June 2025, followed by ATO on 2 July 2025, both of which were well tolerated. By August 2025, her peripheral blood counts had normalized, consistent with hematologic remission.

## 3. Discussion

APL is a subtype of AML typically affecting middle-aged adults (median age 47) with equal sex distribution [2]. APL is characterized by the t(15;17)(q24.1;q21.2) translocation producing the promyelocytic leukemia–retinoic acid receptor alpha (*PML::RARA*) fusion gene. This fusion blocks myeloid differentiation at the promyelocyte stage by preventing RARA from releasing corepressor proteins, silencing the transcription of genes essential for maturation [10].

This results in accumulation of malignant promyelocytes in marrow and blood, causing pancytopenia [11]. This accumulation often triggers coagulopathy via increased promyelocyte-expressed tissue factor, which activates the extrinsic pathway through factor VII binding. Promyelocytes also express annexin II, which accelerates plasmin generation in the fibrinolytic cascade [12]. These changes contribute to disseminated intravascular coagulation and primary hyperfibrinolysis, two of the most dangerous and common early complications of APL [2]. Although most cases involve the *PML::RARA* fusion, there are other APL variants seen. The most frequent variant, *ZBTB16::RARA* t(11;17)(q23;q21), occurs in 1% of APL patients and along with *STAT5B-RARA* t(17;17)(q21;q21) are more resistant to typical ATRA treatment. Other variants such as *NuMA::RARA* t(11;17)(q13;q21), *FIP1L1::RARA* t(4;17)(q12;q21), and *NPM1::RARA* t(5;17)(q35;q21) are more sensitive to typical ATRA treatment [13]. PML::RARA -rearranged variants resemble classical APL morphologically and immunophenotypically, except *ZBTB16::RARA,* which is associated with CD56 expression and treatment resistance [11]. Compared to the variant cases described in the current literature, this case presents morphologically similar to the classical type, and does not appear to be ATO-treatment-resistant, with the patient currently in hematologic remission. The patient’s indolent course remains in stark contrast to the typically aggressive presentation, even among variant cases.

*P53* mutations are rare in APL. However, p53 pathway activity is often decreased because the *PML::RARA* fusion disrupts promyelocytic leukemia protein (PML) nuclear bodies, which are important for p53 activation. ATRA and ATO promote *PML::RARA* degradation and reformation of PML nuclear bodies, restoring p53-dependent senescence essential for leukemia eradication, even though differentiation can occur independently. Rare *p53* mutations occur in therapy-resistant cases, supporting the role of intact p53 signaling in APL treatment efficacy [14]. Notably, the loss of *p53* did not result in a more aggressive initial clinical presentation in this case.

Although there are no reported cases of an indolent de novo APL in a treatment-naïve patient, there are several reported cases of patients with a myeloproliferative neoplasm (MPN) or myelodysplastic syndrome (MDS) who subsequently developed APL following treatment [15,16,17,18,19]. These cases range from aggressive and treatment resistant to slowly progressing and responsive to treatment. Two reports describe essential thrombocythemia (ET) progressing to treatment-related APL [13,14,16,17]. In one case, a *JAK2* V617F-mutated MPN clone persisted in the subsequent APL clone, with poor ATRA/ATO tolerance and severe differentiation syndrome [15]. Another case showed *CALR* type 1-mutated ET evolving into APL with the same CALR mutation [16]. Mamorska-Dyga et al. reported a case of a patient who initially presented with APL (*PML::RARA*, *FLT3*-TKD) but was also found to have a *JAK2* V617F-positive clone, leading the authors to propose that a *JAK2*-mutated MPN clone arose first and later acquired *FLT3*-TKD and *PML::RARA* to transform into APL [17]. Wolach et al. described a case of indolent APL with a smoldering clinical course and responsive to ATRA/ATO similar to our patient; however, unlike our patient, that patient’s APL had transformed from underlying MDS [19].

To our knowledge, a case of indolent de novo *PML::RARA* -positive APL in a treatment-naïve individual has not been previously described. We propose an occult *PML::RARA* -positive clone existed during the initial neutropenia in 2022, with gradual clonal evolution explaining the indolent course. However, the absence of cytogenetic and/or molecular evaluation at that time prevents definitive assessment of this hypothesis. Reported indolent or slowly progressive presentations have involved variant *RARA* fusions or secondary transformation. A few cases documenting disease progression of chronic myeloid leukemia (CML) to a promyelocytic blast phase have been reported [20,21]. In one case, *PML::RARA* -positive promyelocytic cells were detected within two months of initiating tyrosine kinase inhibitor (TKI) therapy, a timeframe considered too early to represent treatment failure; this suggested that a promyelocytic clone had existed prior to therapy and gradually evolved into a fulminant promyelocytic blast crisis. Genetic studies confirmed the presence of both breakpoint cluster region–Abelson (*BCR/ABL1*) and *PML::RARA* fusions [20]. A separate case report described a ZBTB16/RARA variant of APL with an unusually indolent clinical course similar to our patient; however, the variant fusion was also resistant to ATRA treatment [21].

The ETS variant transcription factor 6 *(ETV6)* is crucial for the regulation of hematopoiesis. *ETV6* fusions have been noted in several types of hematological malignancies; it is rare and subsequently under-researched in APL. The available data associates *ETV6* alterations and elevated ETS family expression with poor prognosis and reduced disease-free survival. *ETV6* fusions may inhibit hematopoiesis, resulting in cells remaining in their primitive stage. APL patients with *ETV6* fusions show a significantly higher risk of relapse within one year of remission compared with fusion-negative controls [22].

## 4. Conclusions

This case describes an atypical, indolent presentation of *PML::RARA* -positive acute promyelocytic leukemia in a treatment-naïve patient. The patient presented with intermittent neutropenia over multiple years, followed by recently worsening pancytopenia. Peripheral blood smear showed pancytopenia with marked neutropenia and no circulating blasts, while a suboptimal marrow aspirate revealed rare promyelocyte-like cells and biopsy showed a normocellular marrow with sheets of immature mononuclear cells. Flow cytometry studies detected a small population of blasts with classical immunophenotypic features suggestive of APL. Immunostaining confirmed that the immature cells were diffusely positive for CD117 and myeloperoxidase, with focal CD34-positive myeloblasts corresponding to an area with mildly increased p53-positive staining in a background of CD34-negative cells. The cytogenetic FISH study and next-generation sequencing revealed t(15;17) in approximately 10% of examined cells, p53 loss, a low-level *ETV6* mutation, and the *PML::RARA* fusion.

These findings led to a diagnosis of indolent de novo APL in a treatment-naïve individual, which, to our knowledge, has not been previously described in the literature. APL is generally considered an extremely aggressive subtype of leukemia, although treatment outcomes have improved greatly in recent years. Treatment-resistant cases do still occur in patients with variant *RARA* fusion genes, as well as in individuals with *p53* mutations. However, the patient received induction therapy with all-trans-retinoic acid and arsenic trioxide, which she tolerated well and which led to hematologic remission.

Based on this case, comprehensive morphologic, immunophenotypic, cytogenetic and molecular analysis evaluating for *PML::RARA* variants is recommended, to properly characterize the type of APL and guide treatment plans. Additionally, rare and unusual immunophenotypes of APL, as well as APL mimickers among other AML subtypes, are discussed. APL patients with *ETV6* rearrangements should be monitored closely even after complete remission. This case highlights a novel indolent presentation of APL and emphasizes the importance of recognizing atypical disease courses, as early identification and targeted therapy can achieve remission even in patients with unfavorable molecular profiles.

## Data Availability

The original contributions presented in this study are included in the article material. Further inquiries can be directed to the corresponding author.
